# Intraprocedural MRI-based dosimetry during transarterial radioembolization of liver tumours with holmium-166 microspheres (EMERITUS-1): a phase I trial towards adaptive, image-controlled treatment delivery

**DOI:** 10.1007/s00259-022-05902-w

**Published:** 2022-07-13

**Authors:** Joey Roosen, Lovisa E. L. Westlund Gotby, Mark J. Arntz, Jurgen J. Fütterer, Marcel J. R. Janssen, Mark W. Konijnenberg, Meike W. M. van Wijk, Christiaan G. Overduin, J. Frank W. Nijsen

**Affiliations:** 1 Department of Medical Imaging, Radboud Institute for Health Sciences, Radboud University Medical Center, Nijmegen, The Netherlands; 2 Department of Radiology and Nuclear Medicine, Erasmus University Medical Center, Rotterdam, The Netherlands

**Keywords:** TARE, SIRT, Holmium, Image-guided, Personalization, Dosimetry

## Abstract

**Purpose:**

Transarterial radioembolization (TARE) is a treatment for liver tumours based on injection of radioactive microspheres in the hepatic arterial system. It is crucial to achieve a maximum tumour dose for an optimal treatment response, while minimizing healthy liver dose to prevent toxicity. There is, however, no intraprocedural feedback on the dose distribution, as nuclear imaging can only be performed after treatment. As holmium-166 (^166^Ho) microspheres can be quantified with MRI, we investigate the feasibility and safety of performing ^166^Ho TARE within an MRI scanner and explore the potential of intraprocedural MRI-based dosimetry.

**Methods:**

Six patients were treated with ^166^Ho TARE in a hybrid operating room. Per injection position, a microcatheter was placed under angiography guidance, after which patients were transported to an adjacent 3-T MRI system. After MRI confirmation of unchanged catheter location, ^166^Ho microspheres were injected in four fractions, consisting of 10%, 30%, 30% and 30% of the planned activity, alternated with holmium-sensitive MRI acquisition to assess the microsphere distribution. After the procedures, MRI-based dose maps were calculated from each intraprocedural image series using a dedicated dosimetry software package for ^166^Ho TARE.

**Results:**

Administration of ^166^Ho microspheres within the MRI scanner was feasible in 9/11 (82%) injection positions. Intraprocedural holmium-sensitive MRI allowed for tumour dosimetry in 18/19 (95%) of treated tumours. Two CTCAE grade 3–4 toxicities were observed, and no adverse events were attributed to treatment in the MRI. Towards the last fraction, 4/18 tumours exhibited signs of saturation, while in 14/18 tumours, the microsphere uptake patterns did not deviate from the linear trend.

**Conclusion:**

This study demonstrated feasibility and preliminary safety of a first in-human application of TARE within a clinical MRI system. Intraprocedural MRI-based dosimetry enabled dynamic insight in the microsphere distribution during TARE. This proof of concept yields unique possibilities to better understand microsphere distribution in vivo and to potentially optimize treatment efficacy through treatment personalization.

**Registration:**

Clinicaltrials.gov, identifier NCT04269499, registered on February 13, 2020 (retrospectively registered).

**Supplementary Information:**

The online version contains supplementary material available at 10.1007/s00259-022-05902-w.

## Introduction

Over the past two decades, transarterial radioembolization (TARE) has become an established treatment method, predominantly for hepatocellular carcinoma [1, 2] and colorectal cancer liver metastases [3–5]. Its clinical value and safety have also been explored in cholangiocarcinoma [6] and liver metastases of various other origins, such as breast cancer [7]. Treatment involves injection of microspheres labelled with the beta-emitting isotope yttrium-90 (^90^Y) or holmium-166 (^166^Ho) into the hepatic artery under angiography guidance. The microspheres are transported by the blood flow and lodge in the arterioles in the tumour and healthy tissue because of their size (mean of 30 µm), potentially resulting in high absorbed tumour doses as a result of preferential arterial vascularization of tumour tissue (as opposed to the healthy liver tissue, which is primarily vascularized through the portal venous system) [8]. The treatment parameters such as injected activity and injection positions are partly based on a treatment simulation, and the resulting absorbed dose distribution is only assessed after treatment. As a result, not all tumours necessarily receive an adequate dose and, indeed, many patients have a suboptimal response to TARE [9].

It has become increasingly clear that dosimetry is a key aspect in TARE. A successful TARE treatment is characterized by a high tumour dose and a relative low dose in the healthy tissue. The importance of dosimetry is clearly illustrated in an ancillary study of the SARAH trial, in which it was established that patients of whom all tumours had received a mean dose of at least 100 Gy had a prolonged survival compared to the group that had received less than 100 Gy (median of 14.1 months vs. 6.1 months) [10]. Additionally, in the prospective DOSISPHERE-01 study, it was established that striving for a mean tumour dose ≥ 205 Gy led to an increased response rate of 71% vs. 36% using conventional dose prescription [11]. There is, however, currently no feedback on the actual dose distribution during treatment with TARE, as dosimetry is conventionally only performed after finishing the procedure by means of PET or SPECT imaging.

The ^166^Ho microspheres commercially available for TARE facilitate multimodal imaging after treatment, as they can be visualized and quantified with both SPECT and MRI. The latter intrinsically results in high-resolution dose maps that can be acquired within a relatively short time frame [12, 13]. If a patient would be treated with TARE while positioned in the MRI scanner, MRI quantification of ^166^Ho microspheres could be applied for acquisition of intraprocedural dosimetry, which could be used for an image-guided approach to TARE. In the present study, we investigate the feasibility and safety of performing ^166^Ho TARE within an MRI scanner and explore the potential of intraprocedural MRI-based dosimetry for making TARE an image-guided procedure.

## Methods

### Study design and participants

The EMERITUS-1 study was a single-centre phase I study conducted at the Radboud University Medical Center (Nijmegen, The Netherlands). The primary goal was to evaluate the feasibility and safety of performing ^166^Ho TARE within an MRI scanner. The secondary objective was to explore the capability of intraprocedural MRI-based dosimetry. Eligible patients were of age ≥ 18 years and had a diagnosis of liver-dominant hepatocellular carcinoma, cholangiocarcinoma, (ocular) melanoma, breast cancer or neuro-endocrine tumour. Limited extrahepatic disease was allowed. The minimal life expectancy was 12 weeks or longer, and the WHO performance status was 0–1. Major exclusion criteria were ineligibility for MR imaging (implants, claustrophobia); previous treatment with TARE; radiation therapy of the liver; chemotherapy or major surgery < 4 weeks prior to treatment; serum bilirubin > 26 µmol/L, glomerular filtration rate (MDRD) < 35 mL/min, leukocytes < 4.0 × 10^9^/L and platelet count < 60 × 10^9^/L; and alanine aminotransferase (ALT), aspartate aminotransferase (AST) or alkaline phosphatase (AP) > 5 × the upper limit of normal. A full list of inclusion and exclusion criteria can be found in the Supplementary methods.

All patients provided written informed consent, and the institutional review board committee approved the protocol (CMO Arnhem-Nijmegen, ref. NL68354.091.18). The study was performed in accordance with the Declaration of Helsinki and Good Clinical Practice. Study protocol details were published on clinicaltrials.gov (identifier: NCT04269499).

### Study procedures

All patients underwent a visceral angiography planning procedure in order to pre-identify the catheter positions, coil the gastroduodenal artery if originating close to the injection position and inject technetium-99m-labelled albumin macroaggregates (^99m^Tc-MAA) for treatment simulation. A ^99m^Tc-SPECT was acquired in combination with a low-dose CT directly after the angiography in order to assess tumour uptake, extrahepatic depositions and potential lung shunt. Activity prescription (*A*_60 Gy_) was based on a targeted liver mean absorbed dose of 60 Gy (Eq. 1) as first described in the HEPAR trial [14]. The liver mass (LM) was based on the targeted liver volume (low-dose CT) and a density of 1.06 kg/L. The total activity was divided among the injection positions relative to targeted liver volume per injection position, as identified through cone-beam CT acquired during the pre-treatment angiography.

1 $${A}_{60\mathrm{ Gy}} (\mathrm{MBq})=3781 (\mathrm{MBq}/{\mathrm{kg}})\times \mathrm{LM }(\mathrm{kg})$$

In patient 1, the activity of one injection position was reduced by 50% to prevent toxicity, as the predicted tumour-to-non-tumour (T/N) ratio of the targeted liver volume was low and the targeted region mainly consisted of healthy liver tissue.

**Fig. 1:**
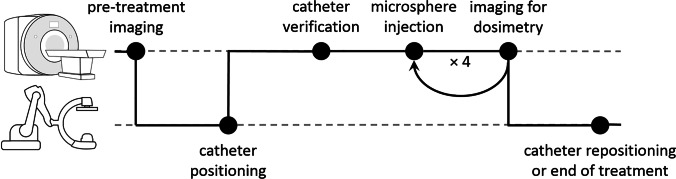
Workflow during the treatment procedure. Top row represents treatment steps at the MRI scanner, and bottom row represents treatment steps at the cone-beam CT. Microspheres were injected in 4 fractions (10% and three times 30% of the microspheres), with imaging for MRI-based dosimetry in between

^166^Ho TARE was scheduled 2 weeks after the pre-treatment angiography. Treatment was performed in a hybrid operation suite, in which a cone-beam CT (CBCT; Artis zeego, Siemens Healthineers, Erlangen, Germany) and a 3-T MRI (MAGNETOM Skyra, Siemens Healthineers, Erlangen, Germany) are located in adjacent rooms with a direct transfer possibility. MR compatibility of all materials and safe usage conditions were investigated in-house prior to the start of study [15]. A schematic outline of the treatment workflow is visualized in Fig. 1. At the beginning of the procedure, T1-, T2- and diffusion-weighted non-contrast-enhanced MRI series were acquired for anatomical reference as well as a holmium-sensitive T2*-weighted series which served as a pre-treatment reference (details on MRI sequences are available in the Supplementary methods). The patient was then transferred to the adjacent CBCT room. For each injection position, the microcatheter was positioned under routine angiography guidance at the location determined during the planning procedure. Finally, the patient was transported back to the MRI scanner to confirm an unchanged catheter position using MRI and injection of microspheres.

^166^Ho microspheres (QuiremSpheres™, Quirem Medical B.V., Deventer, The Netherlands) were injected while the patient was positioned in the MRI scanner, in four different fractions consisting of 10%, 30%, 30% and 30% of the activity determined for the respective injection position. This 10% fraction was designed to mimic a ^166^Ho scout dose [16]. After each injection, a holmium-sensitive MR image series was acquired to capture the microsphere distribution. All four fractions corresponding to 1 injection position were injected within approximately 1 h. After the four fractions had been injected, the patient was transported back to the CBCT, either for relocating the microcatheter to the next injection position or for catheter removal at the end of treatment. Patients were discharged after a 3-h observation period.

The follow-up period lasted for 3 months and included clinical evaluation of adverse events at 1 week, 6 weeks and 3 months post-treatment. Additionally, a ^166^Ho-SPECT/CT (Symbia Intevo Bold, Siemens Healthineers, Erlangen, Germany) was acquired 2 days after treatment, and treatment response was evaluated through contrast-enhanced abdominal CT and/or MRI after 3 months (based on RECIST 1.1 criteria) [17].

### Outcomes

Primary outcomes of this phase I study were the feasibility and safety of ^166^Ho TARE in an MRI environment. The main secondary outcome was the evaluation of intrahepatic ^166^Ho absorbed dose distributions based on MRI during the treatment procedure. Feasibility was evaluated based on the injection positions at which ^166^Ho microspheres could successfully be injected at the MRI, procedure time, the tumours in which dosimetry could be performed based on MRI and the activity recovery ratio (measured activity based on MRI divided by the injected activity). Safety was evaluated through CTCAE v4.0 (serious) adverse event monitoring.

MRI-based dosimetry was performed in a research version of Q-Suite 2.0™ (Quirem Medical B.V., Deventer, The Netherlands), using an in-house developed algorithm for quantifying the microsphere accumulation based on the pre-treatment MRI and the various post-treatment MRIs. Dose reconstruction parameters are available in the Supplementary methods. On a pre-treatment T1-weighted anatomical MRI series, volumes of interest (VOIs) were drawn by a single researcher (JR), supervised by an experienced interventional radiologist (MJA) and a nuclear medicine physician (MJRJ). The healthy liver VOIs were partitioned based on the targeted volume (for instance, right/left hemiliver). Tumour VOIs were mainly drawn for single tumours; however, if tumours were confluent or small and numerous in a single targeted liver volume, multiple tumours were grouped in a single VOI. MRI-based dose maps were manually co-registered to the pre-treatment T1-weighted anatomical MRI in order to evaluate absorbed doses (rigid registration).

### Statistical analyses

Patient characteristics are reported individually as a result of the limited sample size. We calculated descriptive statistics of means and ranges for continuous variables and percentages per category for categorical variables. For analysis of the temporal dosimetry data, a linear trend line was fit to the first three data points of each tumour VOI (after 10%, 40% and 70% of activity had been injected). The fourth data point (after 100% of activity had been injected) was considered different from the linear uptake pattern if deviating ≥ 10% from the expected value. Considering the sample size of this phase I trial, no statistical analyses were performed.

### Role of funding source

The funders of the study had no role in the data collection, data analysis, data interpretation or the decision to submit for publication. The corresponding author had full access to all the data in the study and had final responsibility for the decision to submit for publication.

## Results

### Patient and treatment characteristics

Eight patients were potentially eligible and enrolled in this study. Two patients were excluded from study participation due to hyperbilirubinaemia, meeting the exclusion criteria. A total of six patients were treated with ^166^Ho TARE within the MRI scanner. Baseline characteristics of treated patients are listed in Table 1.

**Table 1:** Baseline characteristics of treated patients

	Patient 1	Patient 2	Patient 3	Patient 4	Patient 5	Patient 6
Age (years)	32	74	43	79	60	81
Sex	Female	Female	Female	Male	Female	Male
Primary malignancy	Breast cancer	Cholangiocarcinoma	Breast cancer	Hepatocellular carcinoma	Colorectal cancer	Hepatocellular carcinoma
Prior treatment	Multiple lines of chemotherapy and hormonal therapy	Gemcitabine/cisplatin	Multiple lines of chemotherapy and hormonal therapy	None	Multiple lines of chemotherapy	None
WHO performance status	0	0	1	1	0	0
Known extrahepatic disease	Locoregional recurrence, axillary lymph node, pleural noduli (2)	Abdominal lymph nodes (2), multiple small pulmonary nodules	Bone metastases (2 vertebrae)	None	Primary tumour (sigmoid) in situ	None
Lab results						
Creatinine (µmol/L)	45	89	74	79	64	88
Bilirubin (µmol/L)	20	4	5	11	13	4
ALT (U/L)	39	30	51	124	10	30
AST (U/L)	80	44	94	96	70	31
Alkaline phosphatase (U/L)	373	175	160	278	187	65
Total liver mass (kg)	4.9 (3.7)^a^	2.0	2.4	2.0	2.4	1.8
Tumour load (%)	62.4	32.4	68.8	16.4	36.2	4.3
Targeted liver part	Whole liver	Whole liver	Whole liver	Right hemiliver	Whole liver	Right hemiliver
Catheter positions	3	2	2	1	2	1
Injected activity (GBq)^b^	8.97	8.06	7.54	4.02	7.88	4.28

^a^As the tumour mass had a large necrotic core (1.2 kg), activity prescriptions were based on a reduced total liver mass.

^b^Injected activity has been corrected for a loss of activity in waste (v-vials, tubing, catheter).

Four whole-liver and two hemiliver treatments were performed with a total of 11 catheter positions (range per patient: 1–3). ^166^Ho treatment characteristics are listed in Table 2. At a 3-month follow-up, two patients had a partial response and four patients had a progressive disease.

**Table 2:** Treatment characteristics

Characteristic	Mean value (range)
Ordered activity (MBq)	7421.1 (4488.1–10,288.8)
Injected activity (MBq)^a^	6789.6 (4015.0–8966.6); 91.4% (87.1–96.4%)
Specific activity (microspheres) (MBq/mg)	12.0 (11.1–14.9)
Holmium content	19.5% (19.1–19.9)
Amount of microspheres (mg)	572.5 (343.2–797.9)

^a^Not all activities were administered as a result of loss in the vials, tubing system and catheter.

### Feasibility

Administration of holmium microspheres at the MRI was feasible in 9 out of 11 injection positions (82%). The first catheter position was not stable enough for transport towards the MRI scanner, as the microcatheter repeatedly dislocated from the left hepatic artery into the right hepatic artery. No other (secondary) dislocations were observed. The second non-feasible injection position was the last catheter position in patient 1, with three catheter positions. This patient was exhausted as a result of the length of the procedure, and therefore, it was decided to complete the procedure at the angiography suite. The mean time per injection at the MRI scanner, including imaging, was 10 min (range 5–19 min). The mean of the total procedure time was 2:29 h:min (range 1:22–3:34 h:min).

At the end of treatment, there was a mean overestimation of administered activity based on MRI of 5.8% (range: − 2.9 to 12.0%) or 452.5 MBq (range: − 115.2 to 964 MBq). This overestimation was relatively consistent between different fractions. The variation between the overestimations in between fractions could be partially explained through a variation in the extent of susceptibility artefacts in the MRI-based quantification near air-holding tissue such as the lungs or gastrointestinal system.

### Safety

All six treated patients were included in the clinical safety analysis, of which all potentially TARE-related data are presented in Table 3. Only two CTCAE grade 3–4 toxicities occurred. The first was abdominal pain in the first week after treatment that responded well to treatment with oxycodone. The second was development of portal hypertension at a 3-month follow-up that required treatment with a beta blocker. No adverse events could be attributed to treatment in the MRI. There were no extrahepatic depositions of the ^166^Ho microspheres.

**Table 3:** Adverse events potentially related to TARE

Adverse event	Any time, *n* (%)	≤ 1 week, *n* (%)	> 1 week, *n* (%)	Grade 3 or 4, *n* (%)
Nausea^a^	5 (83%)	2 (40%)	3 (60%)	0
Fatigue	5 (83%)	0	5 (100%)	0
Abdominal pain	3 (50%)	2 (67%)	1 (33%)	1 (33%)
Back pain	1 (17%)	0	1 (100%)	0
Fever	1 (17%)	1 (100%)	0	0
Weight loss	1 (17%)	0	1 (100%)	0
Portal hypertension	1 (17%)	0	1 (100%)	1 (100%)

^a^Nausea includes vomiting.

### MRI-based dosimetry

At total of 22 tumours were defined across all six patients, of which 3 were treated at the CBCT. Acquired MR images allowed for tumour dosimetry in 18 out of 19 tumours treated at the MRI scanner (95%). In a single tumour close to the diaphragm, dosimetry could not reliably be performed as a result of excessive susceptibility artefacts caused by its close proximity to the lungs.

**Fig. 2:**
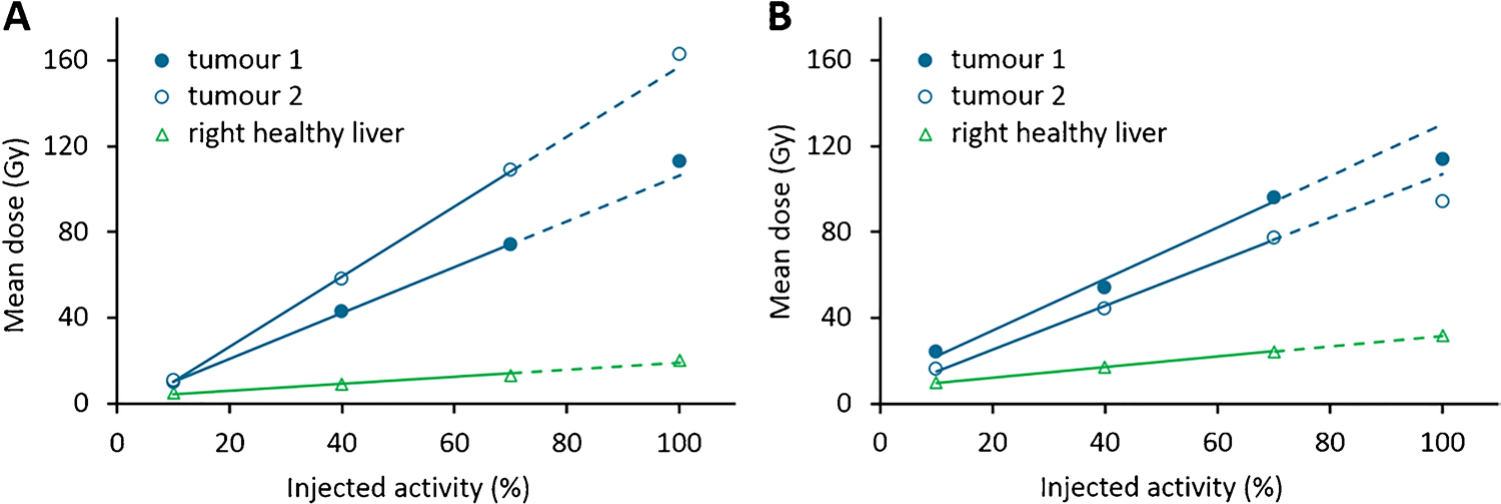
Relationship between injected activity and mean tumour and liver doses of two patients, in whom different uptake patterns were observed (**A** and **B**). Lines are linear trendlines based on the first 3 data points, and the dashed line indicates where the fourth data point is expected based on the linear trend. **A** A patient with hepatocellular carcinoma (patient 4) with a linear increase in mean dose in all volumes of interest. **B** A patient with colorectal cancer metastases (patient 5) with an initial linear increase in mean dose in the tumours (up to 70% injected activity), and then with a decrease in the relative additive dose on the fourth fraction compared to the first three fractions

After administration of all fractions, the mean tumour dose was 78.2 Gy (range: 25.8–125.0 Gy) and the mean healthy liver dose was 29.5 Gy (range: 15.0–44.5 Gy). Corresponding T/N ratios were 1.0–1.5 in 6/22 tumours (27%), 1.6–3.0 in 11/22 tumours (50%) and > 3.0 in 5/22 tumours (23%). The mean dose per tumour per patient over time is reported in Supplementary Tables S1 to S6. The most common uptake pattern was a linear increase in mean tumour dose in all four fractions, which was found in 14 out of 18 (78%) tumours fully treated under MRI. An example of a patient in whom this pattern was visible is presented in Fig. 2A. In one tumour, hardly any uptake was observed, although it followed the linear uptake pattern. The second observed uptake pattern was an initial linear increase in mean tumour dose (first three fractions), with a decrease in the relative additive dose on the fourth measurement compared to the first three fractions. This was observed in 4 out of 18 tumours (22%) in two different patients; an example is drawn in Fig. 2B.

**Fig. 3:**
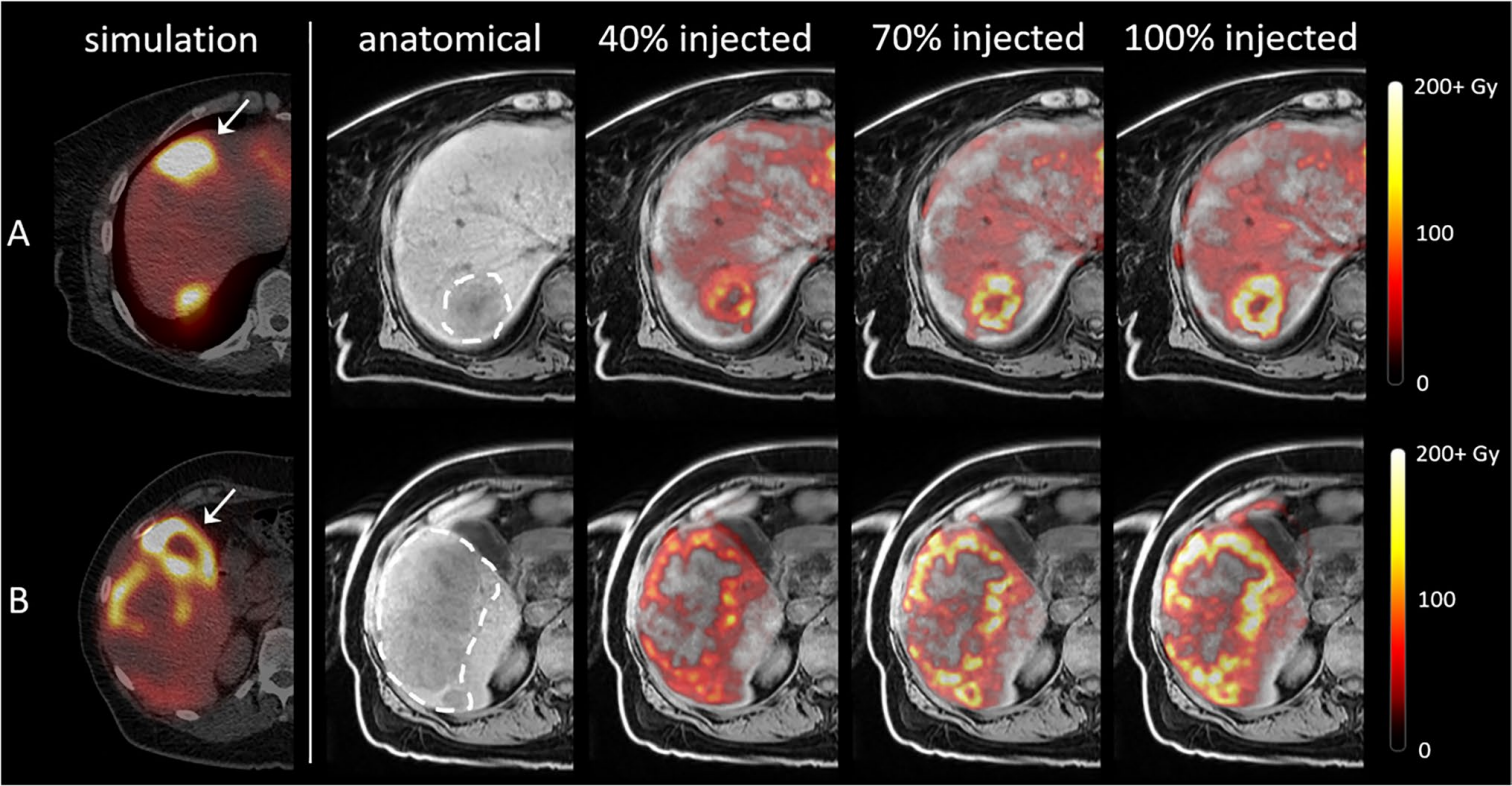
^99m^Tc-SPECT-based treatment simulation and MRI-based dose distributions in patient 5 with colorectal carcinoma liver metastases, in two different slices (rows **A** and **B**). Column 1: treatment simulation after injection of ^99m^Tc-MAA. The high activity deposition in segment IVa and the gall bladder (arrowheads) and low uptake in the dorsal part of the second tumour (row **B**) are probably a result of vasospasm during injection. Column 2: T1-weighted MR images with tumours delineated with the dashed line. Columns 3–5: fusion with MRI-based dose maps generated after 40%, 70% and 100% of the activity had been injected. As more activity is administered, the mean tumour dose increases, but the tumour coverage hardly improves

Dose distributions within tumours were not vastly different between different fractions, as determined through visual inspection. As illustrated in Fig. 3, injection of more microspheres from the same catheter position did generally not result in a more complete tumour coverage.

### Opportunities for image guidance

**Fig. 4:**
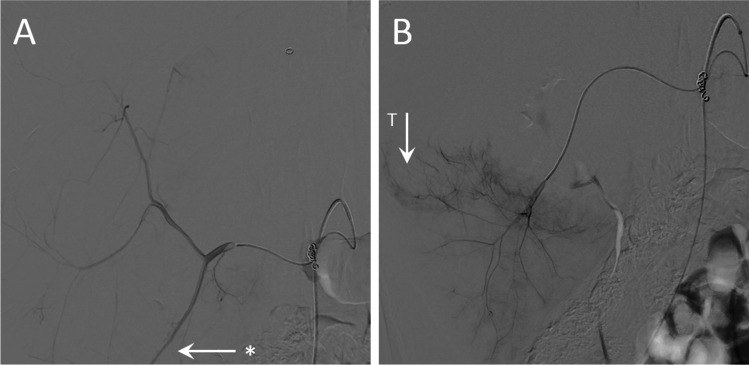
Digital subtraction angiography of the superior mesenteric artery (SMA) of a patient with breast cancer liver metastases (patient 1). **A** The chosen injection position proximal in the SMA. The asterisk indicates a branch of the SMA that is further explored in **B**. **B** More distal branch of the SMA, with contrast enhancement of tumour vasculature (T) that could have been opted for during image-guided TARE

With the ultimate goal of an MRI-guided TARE procedure in mind, we present two cases in which we highlight the found potentially added value of an MRI-guided approach. The first case is the tumour that hardly received any dose, in patient 1, which was vascularized through the superior mesenteric artery (SMA). Digital subtraction angiography (DSA) of this injection position is visualized in Fig. 4A. As this patient presented with extensive breast cancer metastases, the entire liver volume was treated in three injection positions: right hepatic artery, left hepatic artery and the SMA. During pre-treatment work-up, an alternative injection position in a branch of the SMA was considered (Fig. 4B); however, it was decided to treat the SMA region from a more proximal position in order to also reach metastases vascularized through the other sub-branches of the SMA.

**Fig. 5:**
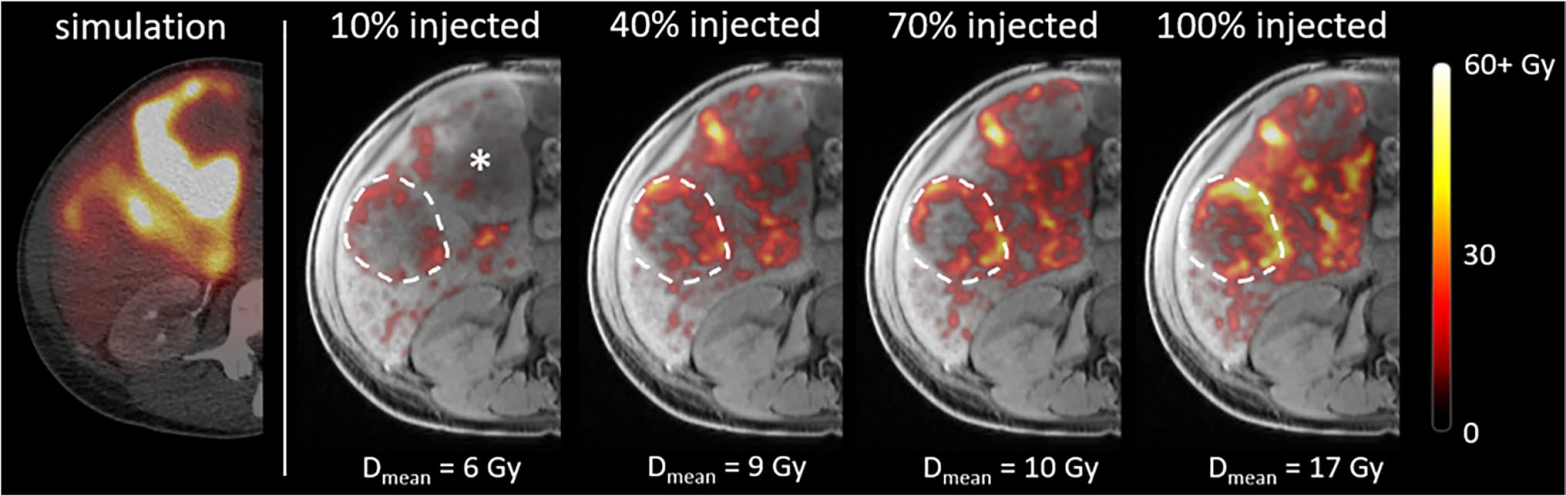
^99m^Tc-SPECT-based treatment simulation and MRI-based dose distributions in a tumour that received a low mean dose (breast cancer metastasis, patient 1) via the superior mesenteric artery (SMA), after different fractions of microspheres had been injected. The tumour is delineated with the dashed line. The asterisk in the leftmost MR image indicates a large, confluent tumour, which was vascularized through both the SMA and the right hepatic artery. *D*_mean_ indicates the mean dose in the delineated tumour

The resulting dose distributions in this tumour are visualized in Fig. 5. After 100% of the planned activity had been injected in this catheter position, a mean dose of only 17 Gy was found, and the posterolateral part of the tumour had remained largely untreated. This tumour targeting could potentially have been improved during an image-guided procedure by relocation of the catheter similar to that in Fig. 4B.

**Fig. 6:**
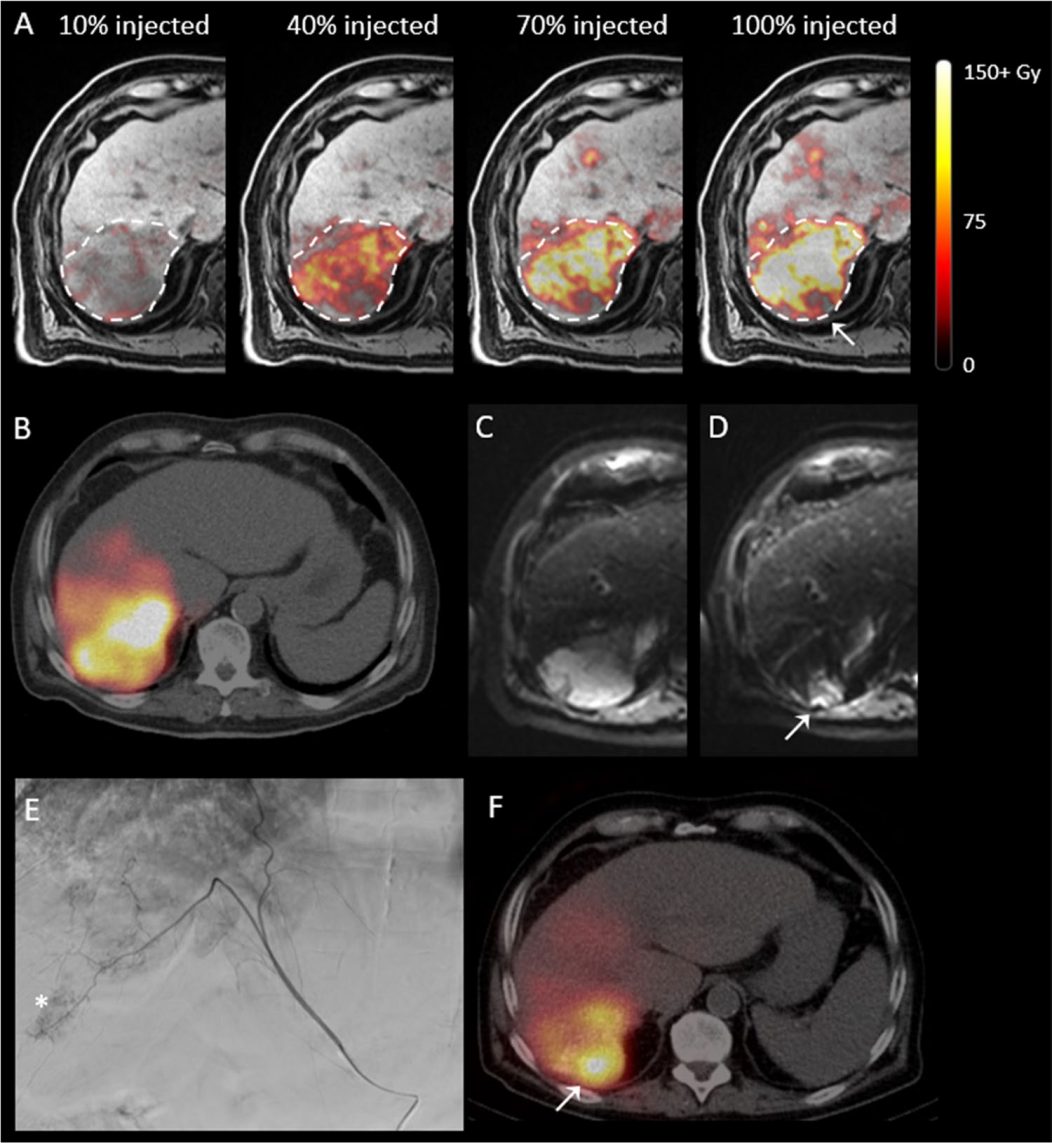
A tumour (hepatocellular carcinoma) of patient 4 treated via the right hepatic artery, in which a small part that was vascularized through an aberrant vessel originating from the phrenic artery remained untreated. **A** MRI-based dose distributions after each of the four fractions of radioactive microspheres had been administered. The tumour is delineated with the dashed line, and the arrow indicates the untreated area. **B** ^99m^Tc-SPECT/CT acquired for treatment simulation. **C** and **D** Diffusion-weighted MRI prior to and 3 months after treatment, indicating persistent diffusion restriction in the untreated area (arrow). **E** Digital subtraction angiography of the aberrant vessel; the asterisk indicates the persistent tumour vasculature. **F **^166^Ho-SPECT/CT after re-treatment of the patient via the aberrant vessel, with high uptake in the untreated area (arrow)

Suboptimal dose coverage was also observed in a hepatocellular carcinoma (patient 4) that was treated via the right hepatic artery. During pre-treatment, an aberrant tumour-feeding vessel originating from the phrenic artery was identified; however, the perfused area appeared to be overlapping with the right hepatic artery. ^99m^Tc-MAA was injected only in the right hepatic artery, and the resulting SPECT/CT indicated complete tumour coverage. It was therefore decided to treat this tumour via the right hepatic artery only. Resulting MRI-based dose distributions are visualized in Fig. 6A.

Even though the mean dose of this tumour increased linearly and the resulting mean end dose was estimated to be 113 Gy, a small part on the dorsal side of the tumour hardly received any dose. This finding was confirmed at a 3-month follow-up, as the suspected non-treated part of the tumour still resulted in diffusion restriction and contrast enhancement on MRI (Fig. 6C, D). The patient was re-treated with ^166^Ho TARE via the right hepatic artery (mean target dose of 40 Gy) and the phrenic artery (mean target dose of 60 Gy), after which the remaining part of the tumour was covered as well (Fig. 6F).

## Discussion

This phase I trial demonstrated the feasibility and safety of performing TARE with the patient positioned in the MRI scanner, allowing for intraprocedural dosimetry. Almost all catheter positions were stable for transport towards the MRI scanner, and MRI-based dosimetry could be performed in 18/19 tumours. No adverse events were attributed to treatment in the MRI. Moreover, we are the first to our best knowledge to acquire dosimetry data during actual TARE treatment delivery and to observe the changing dose distributions as more microspheres are injected.

The majority of the increase in mean absorbed tumour dose (14/18 tumours, 78%) was linearly correlated with the injected activity, which could indicate that most tumours were not saturated with microspheres at the end of treatment. In four out of 18 tumours (22%), we found an uptake pattern that resulted in a lower mean dose than expected based on the linear uptake pattern, albeit only possible to evaluate in the last fraction. This could suggest that these tumours were close to saturation with microspheres, and the maximum achievable mean tumour dose was approached. However, as this was always only the case in the last 30% of the injected activity, this finding may be confirmed in follow-up studies with more injection time points or an increased injected activity. This linear uptake pattern in the majority of the tumours suggests that a small amount of microspheres (such as the commercially available glass ^90^Y microspheres) would result in a similar T/N ratio as a large amount of microspheres (such as the commercially available resin ^90^Y microspheres or the ^166^Ho microspheres). It also further validates the use of ^166^Ho scout dose [16] as a predictor of the final dose distribution, which essentially consists of a small amount of ^166^Ho microspheres. Most importantly, it suggests that, in most cases, the maximum achievable mean tumour dose has not been reached, a maximum which can only be achieved by microsphere saturation of the tumour microvasculature. This leaves a room for personalized treatment optimization, as an increased tumour dose has been correlated with an improved response rate [9]. Using an image-guided approach, more microspheres could be injected until the point of complete tumour saturation, as long as the healthy liver dose remains below the toxicity threshold.

Treatment personalization is one of the key advances to be made to improve treatment efficacy in the upcoming years, not only in TARE [11], but also in other radionuclide therapies such as [^177^Lu]Lu-PSMA [18] or [^177^Lu]Lu-octreotate radioligand therapy [19]. Further personalization of TARE has been recommended by two different expert groups [20, 21], mainly through improving activity prescription based on pre-treatment angiography and treatment simulation findings. Personalizing any form of radionuclide therapy involves adjusting treatment parameters to achieve a sufficiently high tumour dose, while maintaining a low healthy tissue dose. TARE is, however, vastly different from any other radionuclide therapy, as the microspheres have no biological half-life, which reduces the treatment complexity to transferring enough microspheres to the desired target volume, while avoiding deposition in healthy tissue. The microsphere distribution is a dynamic process based on arterial blood flow, catheter positioning and injection technique, as there is no receptor targeting. This is a complex process to predict beforehand, and dose distributions can also be dependent on the injected number of microspheres (i.e. specific activity) and flow redistributions as a result of decreasing flow in the tumour microvasculature. It would therefore be beneficial to have direct feedback on the dose distribution during the procedure. This concept of near real-time intraprocedural dosimetry facilitates a novel approach to TARE personalization, as it does not solely rely on absorbed dose predictions during a planning procedure, but it turns TARE into an adaptive treatment modality that can be adjusted based on intraprocedural findings. Image-guided ^166^Ho TARE is one of the first examples that would fully embrace the theranostic characteristic of the device, in which every former distribution of the radioactive microspheres can be used to predict (diagnose) the distribution of the next therapeutic fraction.

As no definitive dose thresholds have been established that need to be achieved in order to expect a response after TARE [9], an image-guided approach should aim to achieve an as-high-as-possible tumour dose, as long as the healthy liver dose remains low. In healthy pigs, mean absorbed liver doses up to over 100 Gy were relatively well tolerated [22]. A relationship between healthy liver absorbed dose and toxicity has been established in patients with colorectal cancer metastases treated with ^166^Ho TARE, even though no dose-limiting toxicity was observed [5]. The highest found healthy liver mean absorbed dose in that study was 55 Gy, and therefore, it has been recommended as the current healthy liver dose threshold. Similar safety data for patients with cirrhotic livers is expected in the near future from secondary analyses of the HEPAR Primary study [23, 24]. An additional advantage of intraprocedural dosimetry is that the physician can assess not only the mean tumour dose in near real time, but also the coverage of tumours with microspheres through the heterogeneity of the dose distribution. If (part of) a tumour is not reached, the catheter position could be altered empirically (currently with the use of X-ray guidance and cone-beam CT) in order to potentially improve the homogeneity of the intratumoural dose distribution (examples of which have been presented in this study).

Theoretically, imaging modalities other than MRI could also be used for an image-guided approach to TARE. The ^166^Ho microspheres can also be visualized with SPECT, and CT-based quantification has been explored in a preclinical setting [25, 26], in patients with head-and-neck cancer after direct intratumoural injection [27], and has been explored in a single case after TARE [28]. Additionally, different types of ^166^Ho microspheres with a higher density have been developed, which would increase the CT quantification capabilities [29, 30]. ^90^Y-based microspheres used for TARE can be visualized by SPECT [31] and PET [32], and in a recent publication, the CT-based quantification of a novel radiopaque ^90^Y microsphere was investigated in a preclinical model [33]. In general, nuclear medicine imaging modalities have much longer acquisition times than CT or MRI, making them less favourable for image guidance during TARE. MRI-based quantification has an advantage over CT with respect to soft-tissue contrast and is, currently, the most clinically established method [12], but on the other hand, a CBCT-guided approach would have the advantage of being more easily accessible in the hospital and would also facilitate the angiography procedure currently required to position the catheter for injection of microspheres (at the cost of extra radiation dose). All in all, both an MRI-based approach and a CT-based approach currently have their limitations, and research into the further development of either approach is required for full implementation in clinical practice.

The main limitation of our study is the requirement of a rather specific operation room design, and hospitals in which a hybrid OR is positioned directly adjacent to an MRI scanner are scarce. Additionally, our sample size was low, but deemed sufficient to demonstrate the feasibility and potential of an image-guided approach to TARE. Lastly, our current study design was based on using multiple vials of microspheres per injection position. This leads to a loss of activity in the system, as described in an earlier work [34]. An optimal image-guided procedure would use a system depending on only a single vial of activity, to minimize this loss.

Future studies are required to investigate the added value of this image-guided approach, both in terms of additional increase in achievable tumour dose and in patient response. This demands a novel phase I trial of the ^166^Ho microspheres, in which the safety and efficacy of determining injected activity based on intraprocedural dosimetry of healthy liver tissue and tumours has to be established. If an added value of an image-guided approach can be demonstrated and the accessibility of the image-guided approach can be improved, this may enable the ultimate treatment personalization in TARE.

## Conclusion

This study demonstrated the feasibility and safety of a first in-human application of TARE within a clinical MRI system. Intraprocedural MRI-based dosimetry enabled dynamic insight in the microsphere distribution during TARE and illustrated different uptake patterns between tumours. This proof of concept yields unique possibilities to better understand microsphere distribution in vivo and to potentially optimize treatment efficacy through treatment personalization in an image-guided setting.

## Supplementary Information

Below is the link to the electronic supplementary material.

Supplementary file1 (DOCX 24 KB)

Supplementary file2 (PDF 149 KB)

## Data Availability

The datasets generated during and/or analysed during the current study are available from the corresponding author on reasonable request.
