# Supplementary material for: Intraprocedural MRI-based dosimetry during transarterial radioembolization of liver tumours with holmium-166 microspheres (EMERITUS-1): a phase I trial towards adaptive, image-controlled treatment delivery
Source: Eur J Nucl Med Mol Imaging. 2022 Jul 13;49(13):4705–15. doi: 10.1007/s00259-022-05902-w (PMC9606012; doi:10.1007/s00259-022-05902-w)
Supplement: Supplementary file 1 — Supplementary file1 (DOCX 24 KB) [file 259_2022_5902_MOESM1_ESM.docx]

**Supplementary methods**

**List of inclusion criteria:**

1. Patients must have given written informed consent.

2. Female or male aged 18 years and over.

3. Diagnosis of hepatocellular carcinoma or cholangiocarcinoma in the liver or diagnosis of metastatic malignancy to the liver (primary tumours: colorectal cancer, melanoma, breast cancer or neuro-endocrine tumour) with limited disease outside the liver (i.e. liver-dominant disease) defined as the sum of the diameters of all metastases in the liver to be more than 200% of the sum of the diameters of all soft tissue lesions outside the liver.

4. Patient is not amenable for standard therapies (other than TARE) or patient refuses standard therapies

5. Life expectancy of 12 weeks or longer.

6. World Health Organisation (WHO) performance status 0-1.

7. One or more measurable lesions of at least 10 mm in the longest diameter by spiral CT according to the Response Evaluation Criteria in Solid Tumours (RECIST) 1.1 criteria.

8. Negative pregnancy test for women of childbearing potential.

**List of exclusion criteria:**

1. Brain metastases or spinal cord compression, unless irradiated at least 4 weeks prior to the date of the experimental treatment and stable without steroid treatment for at least 1 week

2. Radiation therapy within the last 4 weeks before the start of study therapy.

3. The last dose of prior systemic therapy has been received less than 4 weeks prior the start of study therapy.

4. Major surgery within 4 weeks, or incompletely healed surgical incision before starting study therapy.

5. Any unresolved toxicity greater than National Cancer Institute (NCI), Common Terminology Criteria for Adverse Events (CTCAE version 4.0, see Appendix II) grade 2 from previous anti-cancer therapy.

6. Serum bilirubin > 1.5 x Upper Limit of Normal (ULN).

7. Glomerular filtration rate <35 ml/min, determined according to the Modification of Diet in Renal Disease formula.

8. Alanine aminotransferase (ALT), aspartate aminotransferase (AST), or alkaline phosphatase (ALP) > 5 x ULN.

9. Leukocytes < 4.0 109/l and/or platelet count < 60 109/l.

10. Significant cardiac event (e.g. myocardial infarction, superior vena cava (SVC) syndrome, New York Heart Association (NYHA) classification of heart disease ≥2 within 3 months before entry, or presence of cardiac disease that in the opinion of the Investigator increases the risk of ventricular arrhythmia.

11. Pregnancy or breast feeding (women of child-bearing potential).

12. Patients suffering from diseases with an increased chance of liver toxicity, such as primary biliary cirrhosis or xeroderma pigmentosum.

13. Patients suffering from psychic disorders that make a comprehensive judgement impossible, such as psychosis, hallucinations and/or depression.

14. Patients ineligible to undergo MR imaging.

15. Patients who are claustrophobic.

16. Patient who had prior liver resection and/or coil placement inside the liver, expected to cause imaging artefacts on MRI that will limit MR quantification.

17. Patients who are declared incompetent.

18. Previous enrolment in the present study or previous treatment with radioembolization in the same liver segment(s) as currently requiring therapy.

19. Portal vein thrombosis (tumour and/or bland) of the main branch (diagnosed on contrast enhanced transaxial images). Involvement of the right or left portal vein branches and more distal is accepted.

20. Evidence of untreated, clinically significant portal hypertension (i.e. grade 3 varices at esophago-gastro-duodenoscopy). In these cases, therapy with non-selective beta blocker (propranolol) or rubber band ligation should be instituted according to accepted guidelines. In case of small varices, prophylactic propranolol is advised.

21. Untreated active hepatitis.

22. Transjugular intrahepatic portosystemic shunt (TIPS).

23. Body weight over 150 kg (because of maximum table load).

24. Severe allergy for intravenous contrast agents (Iomeron®, Dotarem or Primovist), depending on the agent used at the time of treatment

25. Lung shunt >30 Gy, as calculated using scout dose SPECT/CT.

26. Uncorrectable extrahepatic deposition of scout dose activity. Activity in the falciform ligament, portal lymph nodes and gallbladder is accepted.

**Supplementary table 1:** Overview of the investigated sequence parameters for MR imaging.

| **Name** | **Sequence type** | **TE/TR (ms)** | **Flip angle (°)** | **Slice thickness (mm)** | **Field of view  (mm × mm)** | **Matrix size** |
| --- | --- | --- | --- | --- | --- | --- |
| T1 VIBE | Spoiled gradient echo | 1.91/4.81 | 6 | 2.5 | 300 × 300 | 192 × 192 |
| T2 TSE | Turbo spin echo | 110/4490 | 150 | 3.0 | 160 × 160 | 256 × 230 |
| T2 HASTE | Half fourier single-shot turbo spin echo | 102/3000 | 180 | 5.0 | 266 × 266 | 256 × 256 |
| TRUFI | Balanced steady state free precession | 2.13/4.26 | 64 | 3.0 | 269 × 278 | 256 × 248 |
| T2*-holmium | Multi-echo gradient echo  (10 echo’s) | 1.06/149 (∆TE: 1.38) | 33 | 4 | 384 × 384 | 192 × 192 |

TE/TR = echo time/repetition time, VIBE = volumetric interpolated breath-hold examination, TSE = turbo spin echo, HASTE = half Fourier single-shot turbo spin echo, TRUFI = true fast imaging with steady-state free precession

**Dose reconstruction**

All multigradient echo (MGRE) image sets were imported into an adapted version of Q-Suite® 2.0 (Quirem Medical B.V., Deventer, The Netherlands). In Q-Suite, a manual segmentation of the liver was drawn and a noise VOI was created in the erector spinae muscle. Within the liver VOI, $R_{2}^{*}$ values were obtained for each voxel, using a mono-exponential fitting method implemented in Q-Suite® (SNR threshold: 2σ, minimum echo’s: 2, S_0_ fit neighbour range: 3, S_0_ fit minimum neighbours: 9).

The in-house developed algorithm that was used for calculating ${\Delta R}_{2}^{*}$ maps from these MGRE images consists of a registration step using an automatic, affine registration followed by a deformable registration using symmetric normalization as the transformation model and mutual information as its similarity metric, as implemented in the SyN algorithm of the Advanced Normalization Tools (ANTs) Python package [1, 2]. After this transformation step, the pre-treatment $R_{2}^{*}$ maps can be subtracted from the post-treatment $R_{2}^{*}$ maps, in order to obtain a ${\Delta R}_{2}^{*}$ map. These ${\Delta R}_{2}^{*}$ maps were then processed in Q-Suite 2.0 as per usual.

**Supplementary references**

1. Avants, B.B., et al., *A reproducible evaluation of ANTs similarity metric performance in brain image registration.* Neuroimage, 2011. **54**(3): p. 2033-44.

2. Tustison, N.J., et al., *The ANTsX ecosystem for quantitative biological and medical imaging.* Sci Rep, 2021. **11**(1): p. 9068.
