# Supplementary material for: Intraprocedural MRI-based dosimetry during transarterial radioembolization of liver tumours with holmium-166 microspheres (EMERITUS-1): a phase I trial towards adaptive, image-controlled treatment delivery
Source: Eur J Nucl Med Mol Imaging. 2022 Jul 13;49(13):4705–15. doi: 10.1007/s00259-022-05902-w (PMC9606012; doi:10.1007/s00259-022-05902-w)
Supplement: Supplementary file 2 — Supplementary file2 (PDF 149 KB) [file 259_2022_5902_MOESM2_ESM.pdf]

Supplementary data

The following tables report the mean dose [Gy] per volume of interest (healthy liver or tumour) over time (injection number), per patient.

Table S1: Dosimetry corresponding to patient 1, who was first treated via the superior mesenteric artery (SMA), then via the right hepatic artery (RHA) and last via the left hepatic artery (LHA). After injection number 6, the patient was exhausted and the remaining two injections were performed at the cone-beam CT (see section results, feasibility). A final MRI was made after the procedure was terminated for the final dosimetry time point. Tumour 2 was vascularized through the SMA and RHA. Tumour 3 was vascularized through the RHA and LHA. Tumour 4 was the tumour in which dosimetry was unreliable, as it was located very close to the lungs (see section results, MRI-based dosimetry).

	**SMA**				**RHA**			**LHA**
injection #	1	2	3	4	5	6	7	8
healthy liver	4	7	9	13	15	15	NA	15
tumour 1	6	9	10	17	19	18	NA	15
tumour 2	3	6	7	10	13	13	NA	28
tumour 3	5	5	5	5	7	11	NA	36
tumour 4	22	20	19	19	39	51	NA	24

Table S2: Dosimetry corresponding to patient 2, who was first treated via the right hepatic artery (RHA) and last via the left hepatic artery (LHA). Tumour 1 was vascularized through both the RHA and LHA.

	**RHA**				**LHA**			
injection #	1	2	3	4	5	6	7	8
healthy liver right	10	22	38	52	51	53	51	53
healthy liver left	6	8	10	8	13	21	31	36
tumour 1	10	29	39	54	57	58	79	91
tumour 2	14	49	76	88	98	100	86	91
tumour 3	12	32	50	70	67	70	68	68
tumour 4	14	36	61	79	80	76	78	88
tumour 5	5	7	10	7	15	24	54	87

Table S3: Dosimetry corresponding to patient 3, who was first treated via the right hepatic artery (RHA) and last via the left hepatic artery (LHA). Tumour 1 was vascularized through both the RHA and LHA (to a much smaller extent).

	**RHA**				**LHA**			
injection #	1	2	3	4	5	6	7	8
healthy liver	3	4	7	10	10	13	19	26
tumour 1	5	21	37	50	52	52	56	61
tumour 2	4	4	6	6	11	30	49	66
tumour 3	2	2	4	8	7	20	23	32

Table S4: Dosimetry corresponding to patient 4, who was only treated via the right hepatic artery (RHA).

	**RHA**			
injection #	1	2	3	4
healthy liver right	5	9	13	20
healthy liver left	4	5	6	6
tumour 1	10	43	74	113
tumour 2	11	58	109	163

Table S5: Dosimetry corresponding to patient 5, who was first treated via the left hepatic artery (LHA) at the cone-beam CT because of an unstable catheter position (see results, feasibility section) and last via the right hepatic artery (LHA). Tumour 3 was vascularized through both the RHA and LHA, and tumours 4 and 5 only through the LHA.

	**RHA**			
injection #	5	6	7	8
healthy liver left	33	34	35	37
healthy liver right	10	17	24	32
tumour 1	24	54	96	114
tumour 2	16	44	77	94
tumour 3	29	41	52	57
tumour 4	66	60	57	59
tumour 5	57	50	54	57

Table S6: Dosimetry corresponding to patient 6, who was only treated via the right hepatic artery (RHA).

	**RHA**			
injection #	1	2	3	4
healthy liver right	10	16	28	38
healthy liver left	6	8	10	7
tumour 1	20	46	81	104
tumour 2	15	56	97	122
tumour 3	18	63	88	149
